# Study of formation of green eggshell color in ducks through global gene expression

**DOI:** 10.1371/journal.pone.0191564

**Published:** 2018-01-29

**Authors:** Fa Qiong Xu, Ang Li, Jing Jing Lan, Yue Ming Wang, Mei Jiao Yan, Sen Yang Lian, Xu Wu

**Affiliations:** College of Animal Science, Fujian Agriculture and Forestry University, Fuzhou, Fujian, People’s Republic of China; Centre de Recherche en Cancerologie de Lyon, FRANCE

## Abstract

The green eggshell color produced by ducks is a threshold trait that can be influenced by various factors, such as hereditary, environment and nutrition. The aim of this study was to investigate the genetic regulation of the formation of eggs with green shells in Youxian ducks. We performed integrative analysis of mRNAs and miRNAs expression profiling in the shell gland samples from ducks by RNA-Seq. We found 124 differentially expressed genes that were associated with various pathways, such as the ATP-binding cassette (ABC) transporter and solute carrier supper family pathways. A total of 31 differentially expressed miRNAs were found between ducks laying green eggs and white eggs. KEGG pathway analysis of the predicted miRNA target genes also indicated the functional characteristics of these miRNAs; they were involved in the ABC transporter pathway and the solute carrier (SLC) supper family. Analysis with qRT-PCR was applied to validate the results of global gene expression, which showed a correlation between results obtained by RNA-seq and RT-qPCR. Moreover, a miRNA-mRNA interaction network was established using correlation analysis of differentially expressed mRNA and miRNA. Compared to ducks that lay white eggs, ducks that lay green eggs include six up-regulated miRNAs that had regulatory effects on 35 down-regulated genes, and seven down-regulated miRNAs which influenced 46 up-regulated genes. For example, the ABC transporter pathway could be regulated by expressing gga-miR-144-3p (up-regulated) with *ABCG2* (up-regulated) and other miRNAs and genes. This study provides valuable information about mRNA and miRNA regulation in duck shell gland tissues, and provides foundational information for further study on the eggshell color formation and marker-assisted selection for Youxian duck breeding.

## Introduction

Green eggshells are not common only in poultry, but also in other avian species and in certain wild birds, such as Eastern birds [1]. The formation of green eggshell color has received attention in the poultry industry. It has been reported that the compoundss biliverdin and biliverdin IX zinc chelate, are detected in green eggshells, while protoporphyrin is detected in brown and light brown eggshells [2, 3]. Biliverdin is a derivative of heme, which results from heme oxygenase-1 (HO-1) activity in the porphyrin pathway and plays an important role in the biochemistry of all living systems [4, 5]. According to the study of Bauer M, Bauer I, biliverdin is formed intracellularly during the degradation of heme in the liver [6], however, recently research proposed that eggshell biliverdin can be directly synthesized in the shell gland and deposited onto the eggshell [7]; the fastest deposition rate at the end of the formation of egg shell [8]. In the final phase of egg formation, the egg enters the shell gland of the parent duck and stays there for 18–20 h to form eggshell. During this time eggshell pigments are secreted into the uterine fluid and progressively deposited in the eggshell, with the deposition rate accelerating in the last 3–5 h before oviposition [9, 10]. The eggshell formation, and the formation of pigment can be observed in the cell lumen at this late point in the process [11]. Therefore, eggs with biliverdin deposited onto the shell exterior will appear green. Biliverdin is synthesized in the shell gland and accumulates in the epithelial cells progressively after ovulation. It has been reported that biliverdin is reduced to bilirubin under the action of biliverdin reductase, then bilirubin glucuronide is synthesized and passed by the bloodstream to the liver where it enters into the duodenum with bile [12]. A small part is reabsorbed by intestinal epithelial cells and returned to the fallopian tube or liver through blood circulation. This deposit is the raw materials and is used to synthesize the eggshell pigment with various enzymes in the shell gland and uterine epithelial cells.

Blue eggshells have been an interesting object for avian genetic studies for a long time. Youxian duck is an important native breed, they not only lay white shell eggs but green shell eggs. Recently, eggshell color has been studied intensely, the main studies performed on shell color have been performed in chicken species [13, 14]; but the molecular mechanisms that control this property are not well understood. The eggshell color is influenced of various factors, including genetic [15], environmental [16, 17] and diet [18]. Researchers have confirmed that shell pigmentation is considered a quality trait that is controlled by multiple genes [15]. For example, Wang *et al*. found that *HO-1* gene is important in the formation of eggshell biliverdin and could lead to blue egg formation when it is expressed highly [19, 20]. The relative expression levels of BLVRA gene have a significantly positive relationship with the reflection coefficient of an eggshell [21]. The genes of *OATP*s, may associated with the transport of eggshell pigments. For example, an EAV-HP insertion could promote the expression of *SLCO1B3* gene in the shell gland to cause the blue eggshell in the chicken [22], and the *SLCO1B3* gene coding a membrane transporter *OATP1B3* which is responsible for transporting amphipathic organic compounds including bile salt [23]. Moreover, it has been found that *ABCG2* can interact with porphyrin and heme structures, and regulates their levels in cells and tissues [24, 25].

Previous studies were focused on *QTL* or several genes, analysis of mRNA or miRNA in eggshell color has not been done in poultry. Therefore, a green-shell-line and a white-shell-line of Youxian duck were bred and they are fifth generation by our team. A global genetic expression approach (RNA-Seq) was applied to shell glands for evaluating and deciphering the transcriptomic profiles of the shell glands to investigate the genetic differences in shell color formation. This technique has been used in other animal species, such as sheep [26, 27] and fish [28]. Gland shell extracted RNAs were submitted to RNA-seq analysis to identify candidate genes that could control the egg shell color. These results should provide a foundation for future studies concerning the genetic and molecular basis of eggshell color formation.

## Material and methods

### Ethics statement

All animal experiments in this study were reviewed and approved by the Institutional Animal Care and Use Committee at the College of Animal Science, Fujian Agriculture and Forestry University. All of the following procedures were strictly performed according to the regulations and guidelines established by this committee.

### Animal material and tissue collection

**Fig 1:**
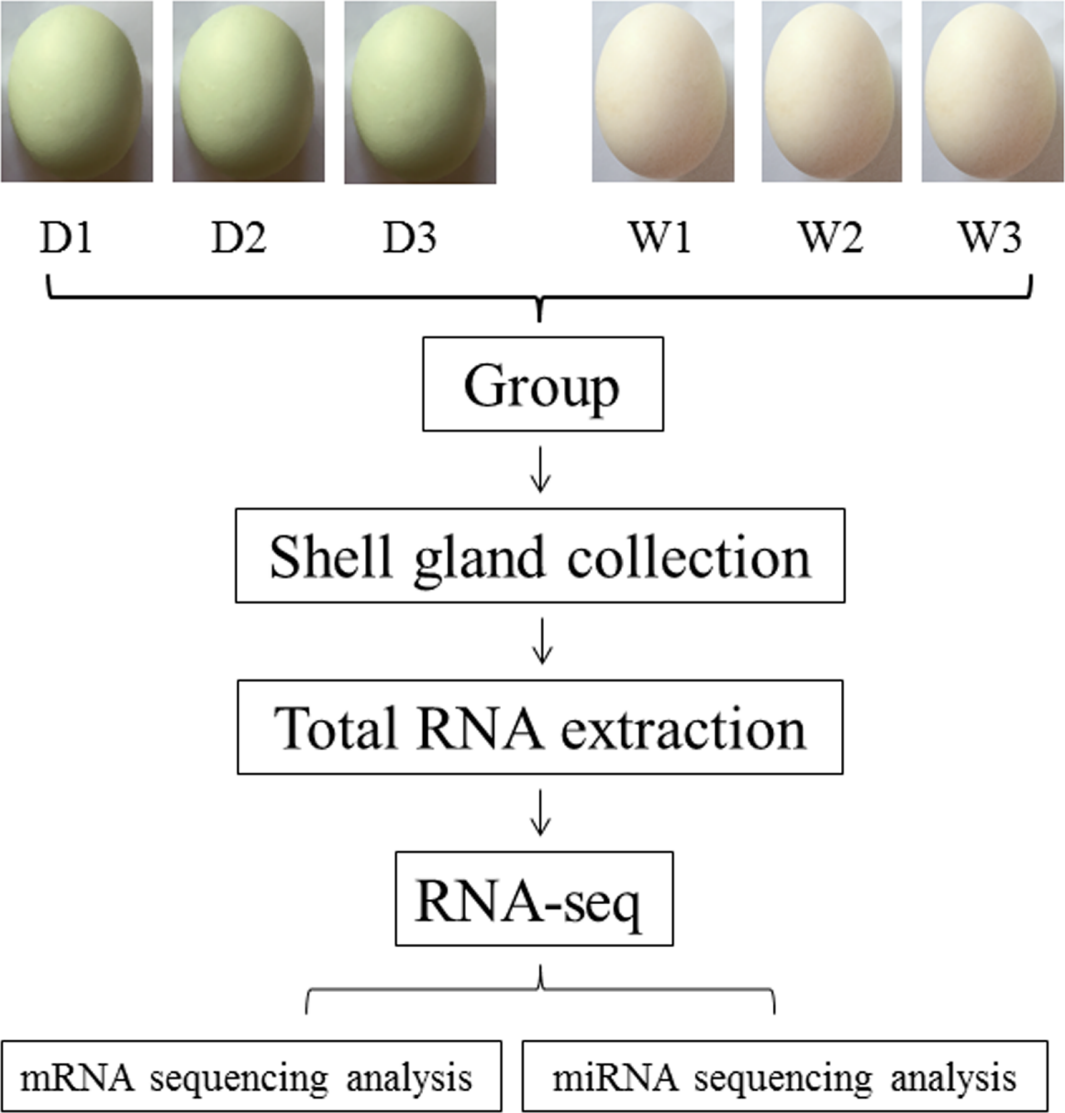
Experimental preparation of shell gland from ducks laying green and white eggs. Note: 1–6 represent D1-D3 and W1-W3 samples; D1-D3 and W1-W3 correspond to samples of three green egg shell glands and white egg shell glands, respectively. M: Trans 2K Plus.

In this study, the Youxian ducks laying white eggs (W) and green eggs (D) were selected into hereditary lines and bred by our team at Guangyang Egg Industry Co. Ltd. China. All experimental ducks were grown in individual cages and were fed with a commercial layer diet. At the 29^th^ week of age, eggs were collected for a week for sampling. The oviposition time of 40 ducks (20 ducks of W and D respectively) was recorded at hourly intervals from 01:00 am to 5:00 am each day. Finally, three ducks of D and three ducks of W with fairly uniform oviposition time were selected for experimental comparison. All ducks were slaughtered after anesthetizing with sodium pentobarbital at 3–5 h before estimated oviposition. The shell glands were harvested after blood perfusion of tissues with ice-cold phosphate buffered saline (PBS) and frozen in liquid nitrogen immediately, then stored at -80°C until the need of total RNA extraction for mRNA and miRNA sequencing analysis (Fig 1) and qRT-PCR.

### RNA extraction, library construction and transcriptome sequencing

Total RNA of shell glands from the two different color of eggshell samples were extracted using TRIzol reagent (Invitrogen, CA, USA) according to the manufacturer’s protocol. RNA degradation and contamination was monitored with agarose gel electrophoresis (S1 Fig). RNA purity was checked using a NanoPhotometer^®^ spectrophotometer (IMPLEN, CA, USA). RNA concentration and integrity were measured and assessed with the Qubit^®^ RNA Assay Kit in a Qubit^®^ 2.0 Flurometer (Life Technologies, CA, USA) and the RNA Nano 6000 Assay Kit with the Bioanalyzer 2100 system (Agilent Technologies, CA, USA) with RIN > 9.0, respectively. A total input amount of 3 μg RNA per sample was used for the RNA sample preparations. Sequencing libraries were generated using NEBNext^®^ Ultra™ RNA Library Prep Kit for Illumina^®^ (NEB, USA) and NEBNext^®^ Multiplex Small RNA Library Prep Set for Illumina^®^ (NEB, USA.) following the manufacturer’s recommendations and index codes were added to attribute sequences to each sample, respectively. Mature mRNA was purified from total RNA using poly-T oligo-attached magnetic beads. The Bioanalyzer was used to assess the library quality, and the effective concentration of the libraries was accurately quantified using qPCR methods. Finally, double-end sequencing of libraries was performed on an Illumina HiSeq™ 2005 platform at the Novogene Bioinformatics Technologies Co., Ltd, Beijing, China. The cDNA samples of shell gland extracts from D and W were sequenced and used to study mRNA and miRNA. Six digital gene expression profiling libraries and six small RNA libraries have been generated from two different shell colors with three biological replicates (three ducks laying green shell eggs line (D1, D2 and D3) and three ducks laying white shell eggs line (W1, W2 and W3). All mRNA and miRNA raw RNA-Seq data were submitted to the NCBI SRA databases with accession number of SRP120128.

### Quality control and reads mapping

Raw reads in FASTQ format were processed with in-house perl, custom perl and python scripts for transcriptome and miRNAs. Next, clean reads were obtained by removing reads that contained an adapter, more than 10% unknown bases ‘N’ and low quality reads from raw data of transcriptome. And clean reads containing unknown bases ‘N’ more than 10%, with 5’ adapter contaminants, without 3’ adapter or the insert tag, containing ploy A or T or G or C and low quality reads were removed from raw data of miRNAs. Then the Q20, Q30, and GC content the clean data were calculated. All the downstream analyses were based on the high-quality clean dataset.

The genome of *Anas platyrhynchos* was used as the reference genome, which was downloaded from the genome website directly as well as gene model annotation files. Index of the reference genome was built using Bowtie v2.2.3; paired-end clean reads were aligned to the reference genome using TopHat v2.0.12 [29]. The small RNA tags also were mapped to reference sequence by Bowtie [30] without mismatch to analyze their expression and distribution compared to the reference genome.

### mRNA expression level analysis

In this study, the read numbers mapped to each gene were counted using HTSeq v0.6.1. The Fragments Per Kilobase of transcript sequence per Million base pairs sequenced (FPKM) of each gene was calculated based on the length of the gene and reads counts mapped to this gene. Pearson correlation between samples was tested by RNA-seq correlativity analysis.

Differential expression analysis of six libraries (three biological replicates per group) was performed using the DESeq R package (1.18.0) [31]. DESeq provided statistical routines for determining differential expression in digital gene expression data using a model based on the negative binomial distribution. The resulting P-values were adjusted using the Benjamini and Hochberg’s approach for controlling the false discovery rate (FDR). Genes with an adjusted P-value of < 0.05 and |Log2 (fold change)| > 1 were found by DESeq and were identified as differentially expressed. Volcano plots, hierarchical cluster analysis and venn charts were used to assess DEGs.

### Sequence data and differential expression profiles analysis of miRNAs

Small RNA tags were mapped on RepeatMasker, the Rfam database or other types of data from the specified species to annotate ncRNA sequences (rRNA, tRNA, snRNA etc.), and then exons and introns of mRNA were aligned to filter and remove degraded fragments. Known miRNAs were confirmed through mapping the reference miRBase20.0 using modified software of Mirdeep2 [32] and Srna-tools-cli and Custom scripts. Novel miRNAs were predicted by integrating the miREvo [33] and mirdeep2 [34] softwares. Mature miRNA expression levels were estimated by transcript per million (TPM) with the following equation [35]: normalized expression = mapped read-count / Total reads_*_1000000. Comparisons between D and W were performed to find significant differentially expressed miRNAs (q-value ≤ 0.05 and |Log2 (fold change)| >1) using the DESeq R package (1.8.3). Hierarchical cluster analysis was performed for differentially expressed miRNAs with similar expression patterns using Cluster3.0 software [36]. Predicting the target gene of miRNA was performed with miRanda [35].

### Functional annotation of DEGs

DEG lists were submitted to the Kyoto Encyclopedia of Genes and Genomes (KEGG) database for enrichment analysis of the KEGG-pathway categories [37]; KOBAS software [38] was used to test the statistical enrichment of differentially expressed genes in KEGG pathways. Target gene candidate genes of differentially expressed miRNAs also were analyzed using KEGG pathway enrichment analysis.

### Interaction analysis of differentially expressed mRNA and miRNA

Based on the target predictions, functional annotation, and negative regulation mechanism of mRNA and miRNA, an interaction analysis of miRNA and mRNA were performed. The miRNA-mRNA interaction networks were displayed using network graph.

### Validation of RNA-seq data by qPCR

To value the reliability of the Illumina analysis, 12 mRNAs and five miRNAs showing differential expression were selected for validation by RT-qPCR. The primers have been listed in S1 Table. After acquiring high quality total RNA, mRNAs and miRNAs were reverse transcribed into cDNA using AMV RNA Kit (TakaRa, Dalian, China) following the manufacture’s protocol. qPCR analyses on the miRNAs and the mRNAs were performed using the Sybr Green PCR Master Mix (TaKaRa, Japan, RR820A) on ABI 7500 Real PCR System (Applied Biosystems) according to the manufacturer’s protocol. The genes *β-Actin* and *U6* were used as internal control for mRNA and miRNA for qRT-PCR, respectively. All RT-PCRs were performed in triplicate and a relative quantitative method (ΔΔCT) was used to evaluate the quantitative variation. The relative fold differences of gene expression were calculated according to the 2−ΔΔCT method.

## Results

### Overview of RNA sequencing results

We sequenced the mRNA and miRNA from total RNA samples. A total of 42.81 gigaytes (GB) of data was generated in the mRNA sequencing results. There were 285,365,190 clean reads filtered from 374,130,794 double-end raw reads in six libraries, with a Q20 and Q30 quality (%) of more than 96.48% and 90.75%, respectively. The GC content of six samples was more than 50.62%. Sequence reads were aligned against the reference genome to obtain 84.84–85.67% of total aligned reads from six samples, of which 59.2–70.7%, 7.8–9.6% and 21.3–31.9% were located in annotated exons, introns, and intergenic regions, respectively (Table 1).

**Table 1:** Basic characteristics of six libraries and sequencing reads mapping to the reference genome.

Sample name	D_1	D_2	D_3	W_1	W_2	W_3
Raw reads	55644086	59818654	64689000	62863866	64115904	66999284
Clean reads	40742200	45541990	55384324	51867956	49504784	42323936
clean bases	6.11G	6.83G	8.31G	7.78G	7.43G	6.35G
Q20 (%)	97.65	96.94	96.48	96.87	97.02	97.55
Q30 (%)	92.86	91.38	90.75	91.34	91.53	92.64
GC content (%)	50.62	51.09	52.39	50.78	51.45	50.62
Total reads	40742200	45541990	55384324	51867956	49504784	42323936
Total mapped	23308345 (57.21%)	26355861 (57.87%)	30526867 (55.12%)	30491933 (58.79%)	28861342 (58.3%)	25880907 (61.15%)
Multiple mapped	811401 (1.99%)	1143844 (2.51%)	646037 (1.17%)	399787 (0.77%)	374604 (0.76%)	495403 (1.17%)
Uniquely mapped	22496944 (55.22%)	25212017 (55.36%)	29880830 (53.95%)	30092146 (58.02%)	28486738 (57.54%)	25385504 (59.98%)
Non-splice reads	13388314 (32.86%)	14801800 (32.5%)	17150842 (30.97%)	17557013 (33.85%)	16503995 (33.34%)	14592012 (34.48%)
Splice reads	9108630 (22.36%)	10410217 (22.86%)	12729988 (22.98%)	12535133 (24.17%)	11982743 (24.21%)	10793492 (25.5%)
Exon	59.20%	65.30%	70.70%	68.70%	68.50%	69.50%
Intron	8.90%	9.60%	8.00%	7.80%	7.90%	8.30%
Intergenic	31.90%	25.10%	21.30%	23.50%	23.60%	22.20%

Note: D-1, D-2, D-3 represents green shell gland; W-1, W-2, W-3 represents white shell gland; Q20: percentage of bases with a Phred value of at least 20; Q30: percentage of bases with a Phred value of at least 30.

The abundance of genes (14090 genes in group D; 14298 genes in group W) was normalized and calculated with FPKM. The distributions of the expression levels of all the genes were similar among the six libraries (S2 Table). Genes with FPKM between 15 to 60 were considered to be expressed at the highest level, while they were shown on at very low levels in the interval 1–3. Considering a threshold of FPKM = 1, 74.68% of the total number of genes were expressed, and there are 627 and 419 genes expressed in W and D, respectively (S2 Fig).

**Fig 2:**
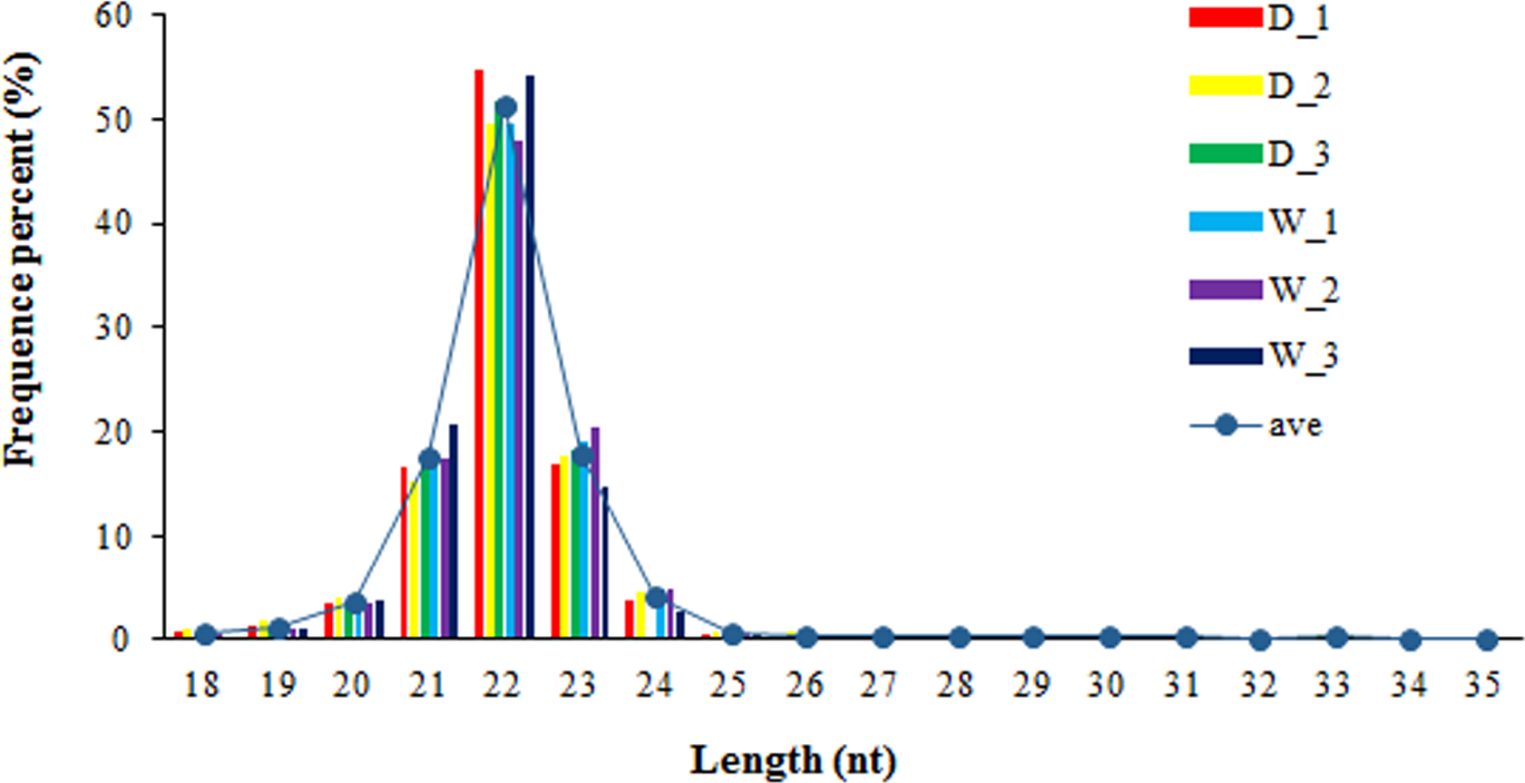
Distribution of sRNA sequence length.

To identify the contribution of miRNAs to pigmentation of egg shell, a deep sequencing method was applied. A total of 10.59 ± 0.15 million (M) and 11.03 ± 0.62 M raw reads were obtained from small RNA libraries of group D and group W, respectively. After remove of the adapters, reads with low quality, 10.32 ± 0.20 M, and clean reads, 10.68 ± 0.56 M, were extracted from group D and group W, respectively (S3 Table). The length distribution of the clean sequences shows little difference in the six libraries, most of the sequences (approximately 90%) being between 21–23 nucleotides (nt) with a distribution peak at 22 nt (Fig 2). After sRNA were mapped to the reference genome (about 84.01% and 80.74%in group D and W, respectively), a total of 260 mature and 195 hairpin RNAs of 262451 known miRNAs, and 199 and 211 mapped mature and hairpin of 210281 novel miRNAs in six libraries.

### Analysis of gene expression level and KEGG pathway by shell color

**Fig 3:**
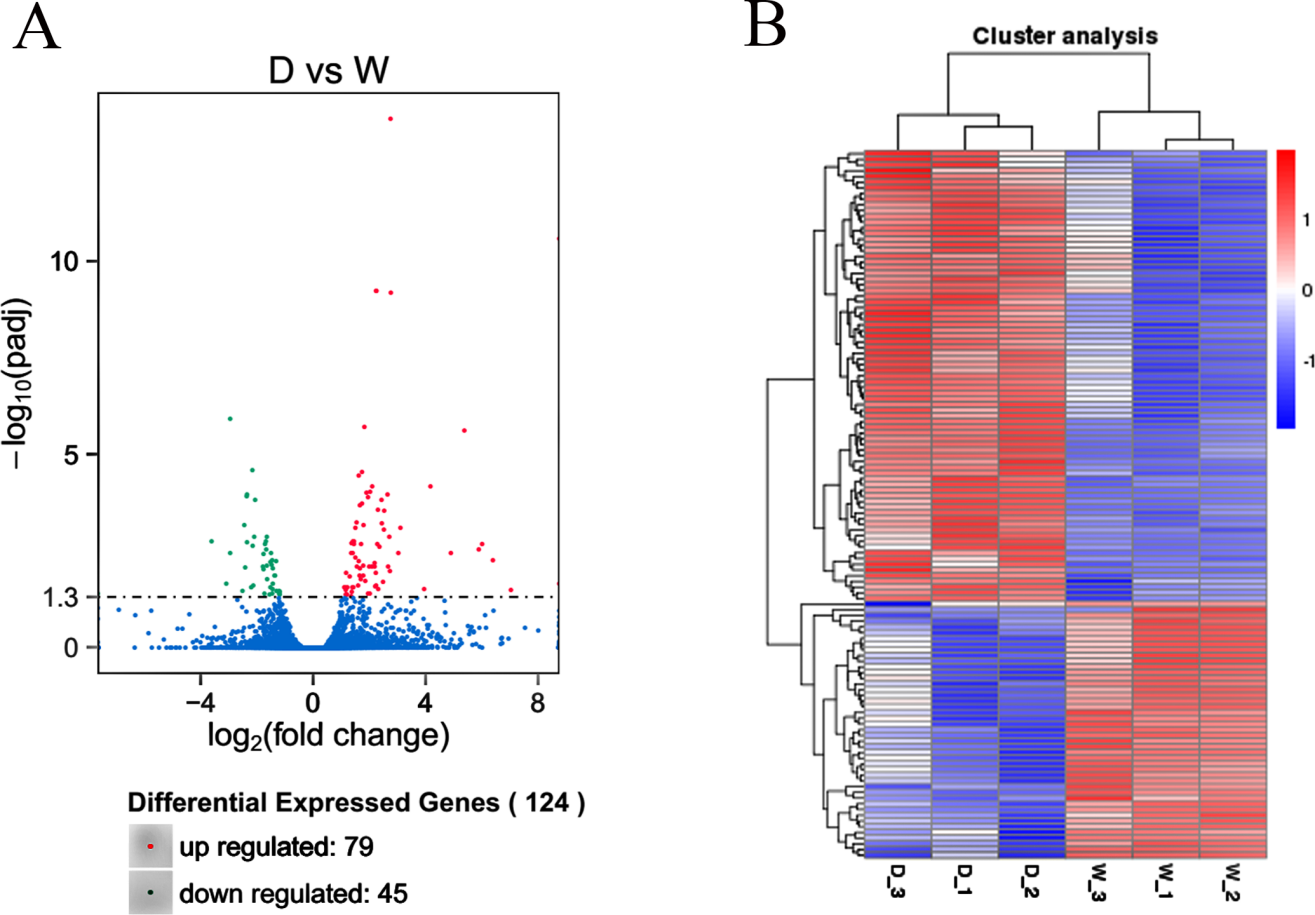
**(A) Scatter plot of DEGs.** Note: Red points represent up-regulated genes with Log2 (fold change) > 0 and q value > 0.05 (-log10 (q value)) ≥1.3); Blue points represent down-regulated genes with Log2 (fold change) < 0 and q value < 0.05 (-Log10 (q value) ≥1.3). Green points represent genes with no significant difference. **(B) Cluster analysis of DEGs.** Note: row: different samples; red and blue represent high expression miRNAs and low expression miRNAs, respectively.

The Pearson correlation between sample gene transcripts were measured before DEGs analysis with R^2^ > 90% in six libraries. This suggests the expression similarity between samples was very close and the sample selection was reliable. A total of 124 DEGs were detected between two shell colors. Analysis of DEGs indicated that there were 79 up-regulated genes and 45 down-regulated genes in group W compared to that observed in group D (S4 Table). The results of DEGs were shown globally on volcano plots and hierarchical clustering was visually displayed (Fig 3). The hierarchical clustering displayed the whole expression of DEGs in W and D with itself special high expression genes clusters.

**Fig 4:**
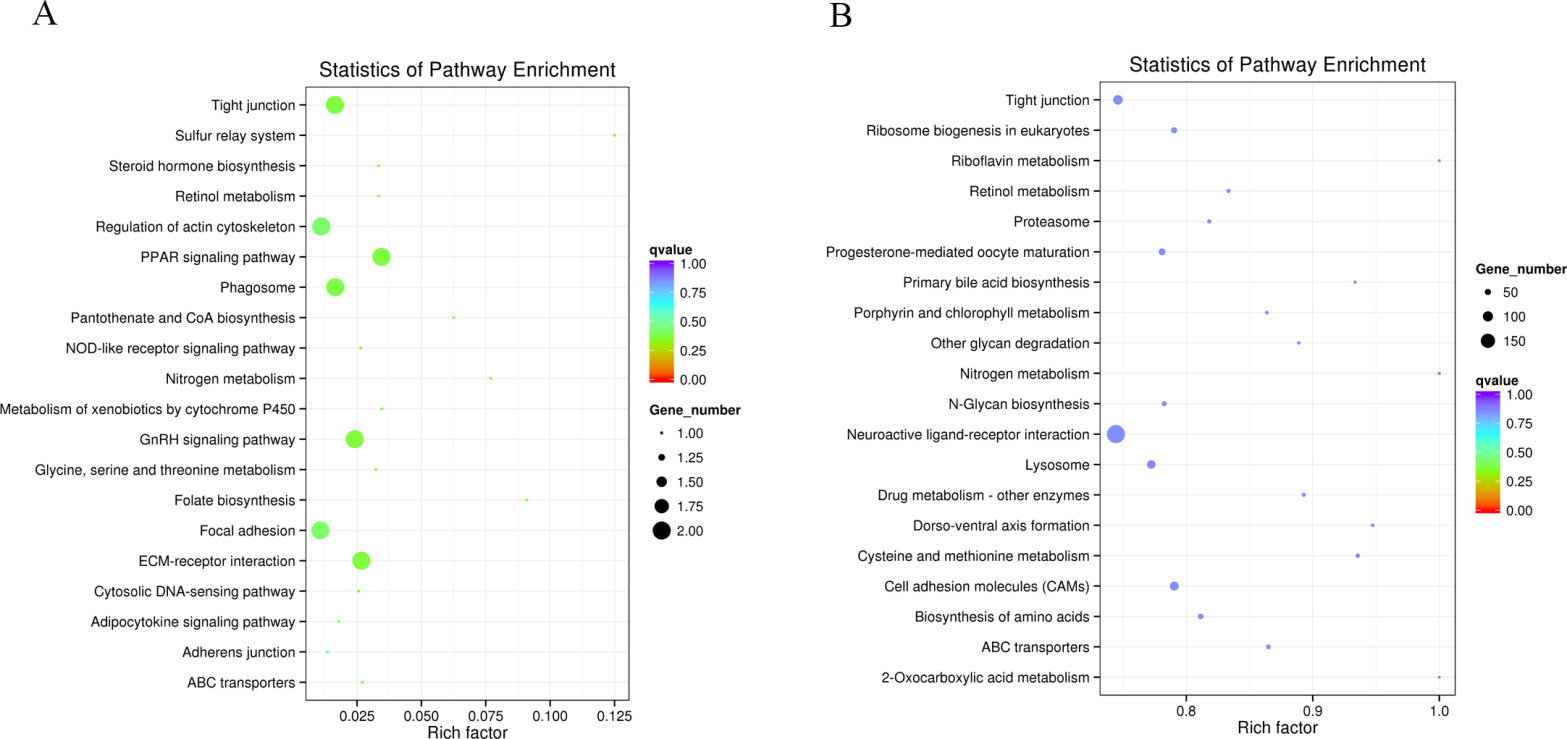
**A. Scatterplot of KEGG enrichment for differentially expressed genes. B. Scatterplot of top 20 KEGG enrichment for target genes.** Note: Rich factor is the ratio of the differentially expressed gene number or target number to the total gene number in certain pathway. The size and color of the dots represent the gene number and the range of the q value, respectively.

**Fig 5:**
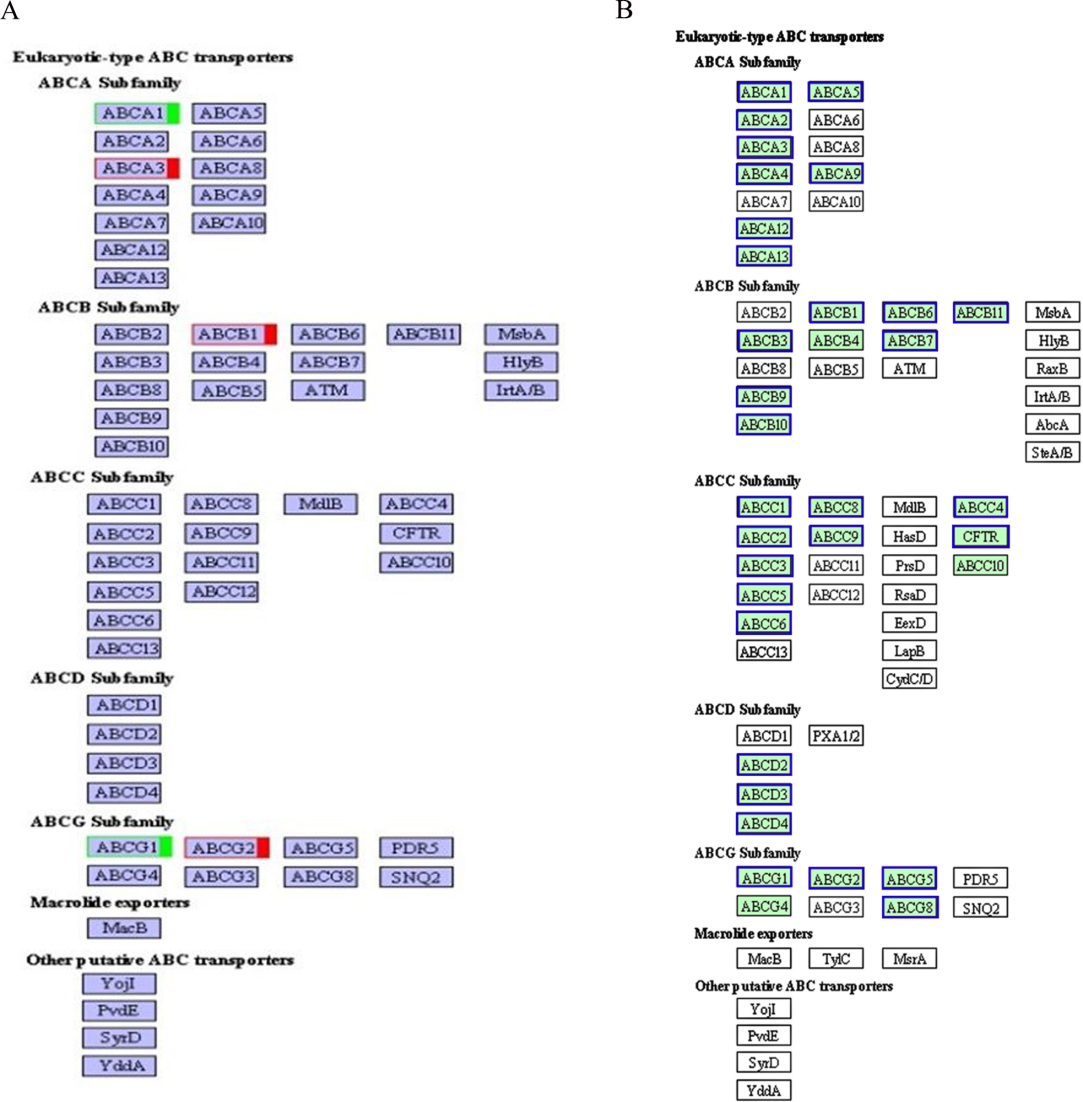
**A. ABC transporter of DEGs.** Note: red represents the up-regulated DEGs, green represents the down-regulated DEGs. **B. ABC transporter of the target genes.** Note: blue represents that the target genes of miRNAs are enriched in this pathway.

Moreover, to further identify related metabolic processes and signal transduction pathways, we performed KEGG pathway analysis by mapping the gene sequences to the reference canonical pathways. Thirty-five KEGG pathways with124 DEGs were identified, including ABC transporters, focal adhesion, the sulfur relay system, etc. (Fig 4A). The most representative pathway related to eggshell color was ABC transporters, followed by regulation of actin cytoskeleton and the PPAR signaling pathway. mRNA sequencing showed that the members of ABCA, ABCB and ABCG sub-families in ABC transporters were differentially enriched, ABCA1 and ABCG1 were down-regulated, while ABAC3, ABCB1 and ABCG2 were up-regulated in group D (Fig 5A). These findings suggest that GEGs might be involved in eggshell color formation.

### Analysis miRNA expression profiling

**Fig 6:**
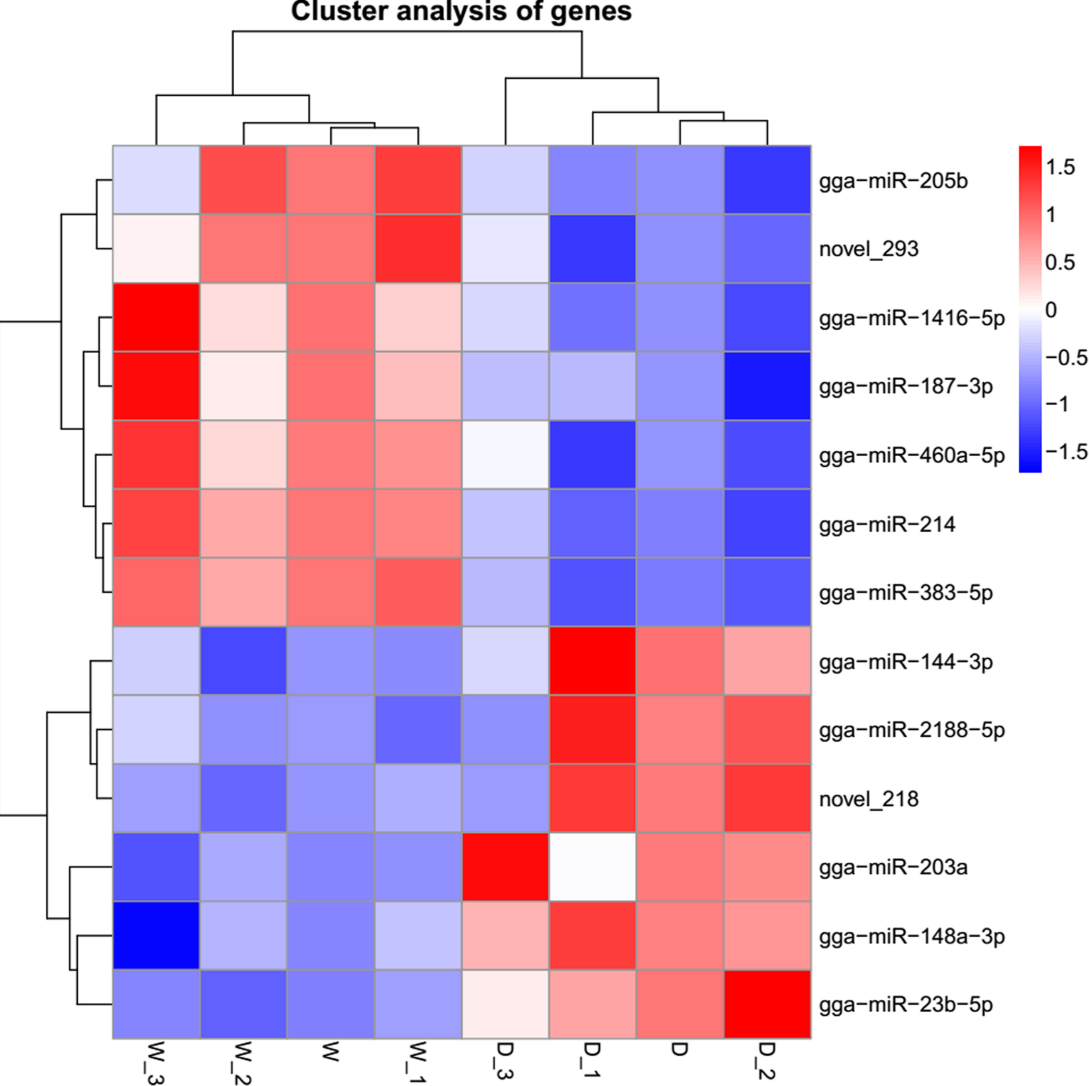
Hierarchical clustering analysis of differentially expressed miRNA. Note: Row: different samples; red and blue represent high expression miRNAs and low expression miRNAs, respectively.

The expression level of miRNA was calculated and normalized to transcripts per million (TPM), which the expression at the highest level was the interval 0.3–3.57 in each libraries (S5 Table). The results of Pearson correlation suggest that the expression similarity between samples was very close and the sample selection was suitable (R^2^ > 96%). We analyzed differentially expressed miRNAs between two different color with filter criteria padj < 0.05 and |Log2 (fold change)| > 1. Six up-regulated and seven down-regulated differentially expressed miRNAs in group D were identified (S6 Table) and the hierarchical clustering analysis shown is shown in Fig 6. Among them, the largest fold change was gga-miR-144-3p; and the least of q value was gga-miR-203a. In order to further study the functions of miRNAs, 262451 and 210281 target genes of known miRNAs and novel miRNAs were predicted in this experiment, respectively, in which a hundred miRNAs interacted. For example, within gga-let-7a-2-3p, gga-let-7c-3p, gga-miR-148a-5p, gga-miR-148b-3p etc., 44 miRNAs can regulate the same target gene (S7 Table). The same miRNA also can target by hundred genes, such as gga-miR-144-3p which can targeted with 958 genes (S7 Table).

After target prediction, KEGG pathway analysis of the target genes was conducted. In total, the target genes of 13 differentially expressed miRNAs were enriched in 154 KEGG pathways. According to KEGG function annotations, we classified the predicted target genes to find the pathways that were actively regulated by miRNAs in shell gland tissue. Each pathway was annotated by many target genes, for example 168, 139, 199, 35, 172 and 5 target genes were mapped to the regulation of actin cytoskeleton, protein processing in endoplasmic reticulum, MAPK, ABC transporters, focal adhesion and phenylalanine, tyrosine and tryptophan biosynthesis pathways, respectively. This finding indicates that the miRNA target genes may be related to protein processing, transmembrane transport, amino acids generated and other biological process in the formation of eggshell color. Interestingly, we found several pathways, regulation of actin cytoskeleton, the adherens junction, the PPAR signal pathways and ABC transporters, which were listed in top 20 in the comparison of miRNA and mRNA transcriptome (Fig 4B). Noticeably, miRNA target genes were enriched in ABC transporters (Fig 5B), including ABCA, ABCB, ABCC, ABCD and ABCG subfamilies, which implied miRNAs can affect the function of ABC transporters by regulating target genes and subfamily interaction during eggshell color formation.

### Interaction analysis of differentially expressed mRNA and miRNA

**Fig 7:**
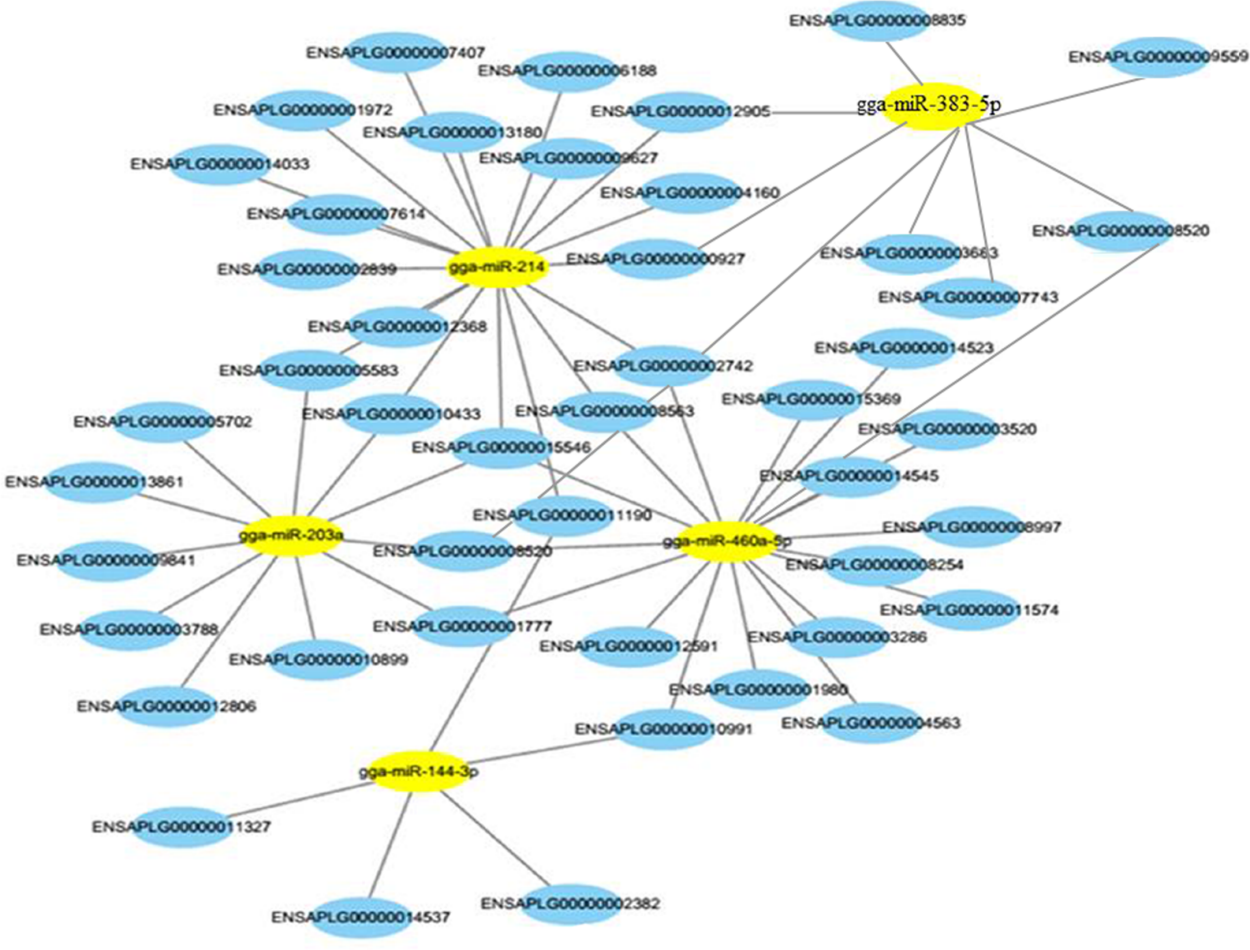
Functional regulation networks of five differentially expressed miRNAs and respective target genes. Note: yellow represents core differentially expressed miRNAs; blue represents target genes. (Several important genes: *ENSAPLG00000014523*: *FABP7*, *ENSAPLG00000004563*: *CD36*, *ENSAPLG00000008520*: *SLC12A8*, *ENSAPLG00000002382*: *ABCG2*).

We further integrated analysis the differentially expressed genes and miRNAs data into the predicted miRNA-mRNA pairs to validate the target pairs. The interaction between 124 DEGs and 13 differentially expressed miRNAs were analyzed, which showed that 59 DEGs (37 up-regulated mRNAs and 22 down-regulated mRNAs) can be regulated by 13 differentially expressed miRNAs (6 up-regulated miRNAs and 7 down-regulated miRNAs). The interaction networks of the miRNA-mRNA pairs are illustrated in S3 Fig. To further define the functional characteristics of miRNA-mRNA interaction pairs, KEGG pathways analysis was done for candidates target genes and DEGs. A total of 24 signal pathways were identified including five significantly enriched pathways using P-value < 0.05 as a threshold, such as ABC transporters, PPAR signal pathways (S8 Table). The miRNA-mRNA interaction network is complex and it is important to establish the roles for specific miRNAs or miRNA-mRNA interactions in eggshell color formation. For instance, gga-miR-144-3p, gga-miR-460a-5p and gga-miR-203a constituted a complex interaction network with FABP7 (FABP), CD36, ABCG2 and many other miRNAs and mRNAs (Fig 7). Furthermore, two target genes (FABP7 and CD36) are significantly enriched (P-value < 0.05) in PPAR signal pathways (S4 Fig).

### Validation of differentially expressed mRNA and miRNA

**Fig 8:**
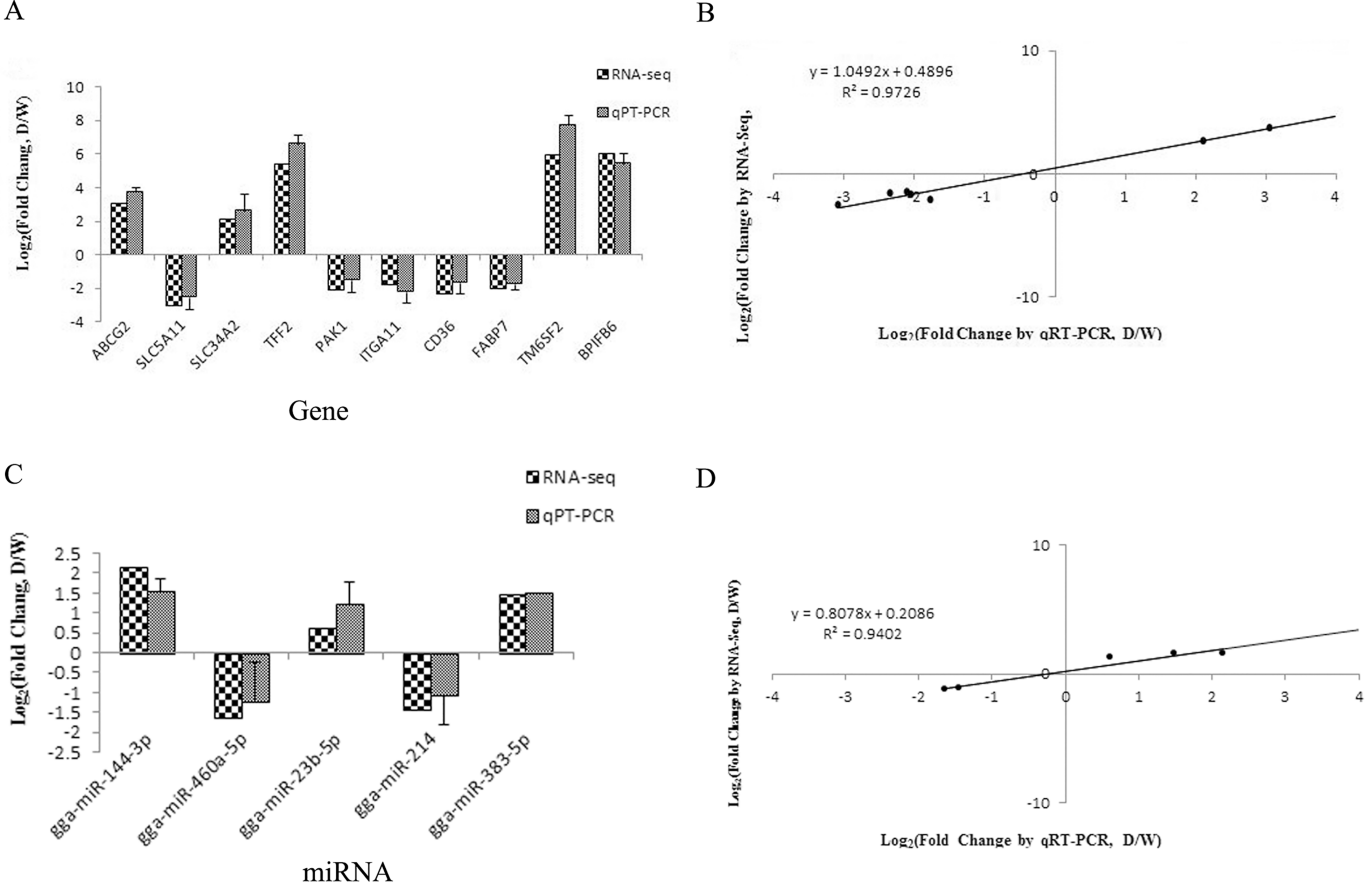
(A) Comparison of DEGs fold changes between RNA-seq and qRT-PCR; (B) Pearson correlation scattered plot comparison of RNA-seq based and qRT-PCR based results; (C) Comparison of differentially expressed miRNAs fold changes between RNA-seq and qRT-PCR; (D) Pearson correlation scattered plots comparison of differentially expressed miRNAs in RNA-seq based and qRT-PCR based results.

To further verify our sequencing data, we analyzed 12 DEGs and five differentially expressed miRNAs by qRT-PCR. The mRNA verification results are shown in Fig 8A, and the qRT-PCR expression trend was in accordance with the RNA-Seq result. The curve fit is shown in Fig 8B with a correlation value was 0.9726, which accounted for the high correlation between results obtained by RNA-seq and qRT-PCR. From the miRNA verification result (Fig 8C) and curve fit (Fig 8D), we can visualize the expression level of qRT-PCR was in correlation with RNA-Seq. In conclusion, the results of qRT-PCR were in line with RNA-seq analysis, indicating that RNA-seq data were relatively reliable and the the RNA-seq method could be used to identify differentially expressed miRNAs and genes.

## Discussion

### Analysis of DEGs for pathways

A total of 124 DEGs including 79 up-and 45 down-regulated genes were identified and they involved in multiple pathways in this study. This could provide important statistics to reveal the regulatory mechanism network of green eggshell formation. Numerous studies suggest that the formation of eggshell color is regulated by many genes rather than a single gene [39, 40], which is in accord with our results. Interestingly, *ABCG2* is one of the DEGs from the shell gland of Youxian duck green eggshell line which is up-regulated expression in its gene family. *ABCG2* is a member of the ABCG subfamily, also known as adenosine triphosphate binding cassette transporter G2, mainly located in the cell membrane, which regulates the expression of endogenous protoporphyrin [41]. Furthermore, we also found some DEGs from ABCB, ABCA, ABCC, ABCD subtribe, such as *ABCB1*, it has been identified as potential unconjugated bilirubin transporters, which export the pigment from the cells [42]. *ABCB1* expressed up-regulation in our results, which could explain that ABCB subtribe is important for the sources of eggshell pigment and other related processes. Studies has suggested that *ABCB7* plays a role in heme biosynthesis [43] and could result in anemia if it expresses exception [44]. *ABCB1* and *ABCB7* belong to the same subtribe and have similar features, in which normal expression may influence the heme synthesis. These genes belong to ABC transporters super families, which can transport endogenous and exogenous transmembrane substances and metabolites of structural diversity and has significant effects on the absorption and treatment of chemicals in the body. ABC transporters that effect coloration have been discovered in *D*. *melanogaster* [45] and silkworms [46]. They can transport multifarious endogenous and exogenous substances and metabolites, which are active in the plasma membrane and intracellular endothelium in small molecule transport. Similarly, we can hypothesize that there are some ABC transporters responsible for transportation of pigment-related substrates to influence pigmentation in egg shells. Above all, we supposed the ABC genes (*ABCG2*, *ABCB1* and *ABCB7*) showed a promising influence on the formation of green shell color, also suggesting that gene regulation for eggshell color is a complex network.

OATPs, are numbers of SLC supper family, are another important and interesting metabolism pathway in this study. These kinds of membrane proteins mediate various substances transportation in the cell membrane, and biliverdin is one of the transshipment substrate of OATP. In this study, five members of SLC, such as *SLC28A3* and *SLC5A11*, are identified in the DEGs. We inferred that the interaction of SLC DEGs involved in the formation and transportation of pigment or small molecular transport in eggshell color's formation. Recently, Wang *et al*. found that EAV-HP is closely associated with overexpression of *SLCO1B3* in the Dongxiang chicken; the blue eggshell phenotype is correlated with ectopic expression of *SLCO1B3* in shell glands of the uterus using *in situ* hybridization [22]. As indicated in our inference, the expression of SLC family members at a high level in the shell gland is associated with the green shell phenotype. *SLC28A3* can transfer pyrimidine nucleosides as well as purine nucleosides [47] to participate in the metabolism of amino acids. Damaged adenine nucleotide transport genes resulted in a reductions of heme synthesis in yeast [48], due to a lack of synthetic materials for heme derived from glycine and succinate. Heme is an important precursor to the main pigment in green eggshell and is significant for the formation of eggshell pigment. Together, the shell color will become darker with increased expression levels of *SLCO1A2* and *SLCO1C1* [49]. These reports agree with our result that SLC members participate in the formation of pigment. In addition, our results also show that although the DEGs come from one family, the expression of each member may be different or selective in different varieties.

### Analysis of miRNAs and target genes

In our study not only were DEGs identified, but multiple differentially expressed miRNAs were characterized. Our study identified multiple novel miRNA molecules (such as gga-miR-144-3p, gga-let-7a-3p and gga-let-7a-5p) that might be significant in the formation of green-shell eggs. At present, the identification of miRNA affected eggshell color has been rarely reported. In this study, gga-miR-144-3p, belonging to the miR-144 family, expressed at high level in the shell gland of green shelled egg so miR-144-3p is speculated to be involve in the formation of the pigment. Recent studies suggested the miR-144 and miR-451 can accelerate the differentiation of hematopoietic stem cells and hinder their growth among other effects [50]. Moreover, the shell pigments were considered to originate the decomposition of red blood cells in the fallopian tube mucosal layer through the uterine epithelium. Most interestingly, gga-miR-144-3p is significantly differentially expressed in D group.

Ten other known miRNA and 11 novel miRNA together targeted *ABCG2* gene. The other subfamily of ABC transporters also enriched except for the ABCG subfamily. Those results suggest that gga-miR-144-3p can regulate *ABCG2* expression to play a role in egg shell color formation. Another differentially expressed miRNA between green shell and white shell glands is gga-let-7a-3p. miRNAs gga-let-7A-2-3p and gga-let-7c-3p can control of *SLC5A11*, which proves let-7 indeed affects the formation of egg shell color. It was found that three miRNAs (let-7, let-7-5p and let-7k) and four miRNAs (let-7i, let-7g, let-7a and let-7d) were highly expressed in the feather follicles of ducks and the skin of mice, respectively; let-7b, let-7c and let-7 expressed both in two animals [51]. This suggests that miRNA from the same family may be involved in a variety functional regulation. Our results enrich the miRNA database of Youxian duck and hve significance in the study of miRNA function while laying the groundwork the molecular mechanism of egg shell color formation.

### The association analysis of differentially miRNAs and genes

Integrative analyses of mRNAs and miRNAs by RNA-Seq may be conducive to understanding the genetic regulation network that participate regulation of eggshell color formation. It was important for us to explore the mechanism of eggshell color formation. Researchers have recently created an network of miRNA-mRNA of adenocarcinoma regulation, and found that miR-106b, miR-191, miR-19b, miR-92a, miR-92b, miR-93, and miR-141 are key elements [52]. Similarly, in our study, gga-miR-144-3p, gga-miR-460a-5p, gga-miR-214 and gga-miR-383-5p could interact with *ABCG2*, *FABP7*, *CD36* and *SLC12A8* to regulate ABC transporters, the SLC pathway and the PPAR pathway in the interaction network in the formation of egg shell pigment. gga-miR-144-3p (up-regulated in D group) which differentially expressed between green shell glands and white shell glands, targeted with *ABCG2* (up-regulated in D group) in the regulation network. The ABCG2 protein is considered to be a key enzyme that transfers protoporphyrin out of cell, it has been reported that *ABCG2* can interact with heme structures and protoporphyrin to prevent them from depositing in cells and tissues [53]. Through a series of reactions protoporphyrin IX can be converted to biliverdin, biliverdin is thought to be related to the formation of green shell eggs. This suggests that this gene is associated with green shell coloration, which may provide material for biliverdin synthesis. De Aguiar *et al*. showed that the enhancement of the expression level of microRNA-144 can reduce the expression level of ABCA1 protein in the liver [54]. Furthermore, gga-miR-460a-5p down-regulated *FABP7* and *CD36* in the D group of our study in the PPAR signal pathway (the most significant enrichment of 24 identified pathways). It is reported that *CD36* plays a role in proximal absorption of dietary fatty acid and cholesterol for optimal chylomicron formation [55], and in the transport of fatty acids across the human brain micro-vessel endothelial cells [56]. *FABP7* is suggested to be involved in the proliferation of astrocytes by controlling cellular fatty acid homeostasis [57]. Therefore, *FABP7* and *CD36* might be involved in metabolic activity of pigmentation, molecular transport and energy metabolism. Altogether, this suggests that integrative analysis of mRNAs and miRNAs not only enrich the regulatory mechanism of eggshell pigment formation, but are powerful tools to identify novel miRNA-mRNA regulation networks.

## Conclusion

This study is the first investigation of miRNA and mRNA related to the formation of green eggshell color in poultry. We identified differentially expressed mRNAs and miRNAs related to eggshell color formation between white eggshell line and green eggshell producing Youxian duck lines. In addition, pathway analysis demonstrated that numerous important DEGs are involved in ABC transporters and SLC pathways. Moreover, interaction analysis of mRNA and miRNA showed that miRNAs can regulate eggshell color formation by regulating cellular pathways, for example, in ABC transporter, *ABCG2* could be up-regulated by gga-miR-144-3p. These findings provide fundamental molecular resources to conduct further studies on the mechanism of eggshell color formation.

## Supporting information

S1 Fig Distribution of sRNA sequence length. (TIF)

S2 Fig Comparison of the identified genes between W and D. (TIF)

S3 Fig Regulation network of differential expressed miRNAs and mRNAs. Note: Square nodes and round nodes in the figure represent miRNAs and genes, respectively. The line represents the correlation between the differentially expressed miRNA and target genes. Red and green represent up and down differential expressed miRNAs and mRNAs respectively.
(TIF)

S4 Fig PPAR signal pathway. Note: red represents target genes that are enriched in the pathway.
(TIF)

S1 Table Primers for mRNA and miRNA detection by real-time RT-PCR. (XLS)

S2 Table Number of genes in different FPKM ranges. (XLS)

S3 Table Summary of common and specific sRNA sequences between any two libraries. (XLSX)

S4 Table List of differentially expressed genes. (XLS)

S5 Table Number of miRNAs in different TPM interval. (XLS)

S6 Table Results of differentially expressed miRNA. (XLSX)

S7 Table Target genes of differentially expressed miRNAs (example). (XLS)

S8 Table Pathway analysis of interaction mRNA and miRNA. (XLSX)
