# Supplementary figures and images for: Study of formation of green eggshell color in ducks through global gene expression
Source: PLoS One. 2018 Jan 29;13(1):e0191564. doi: 10.1371/journal.pone.0191564 (PMC5788541; doi:10.1371/journal.pone.0191564)

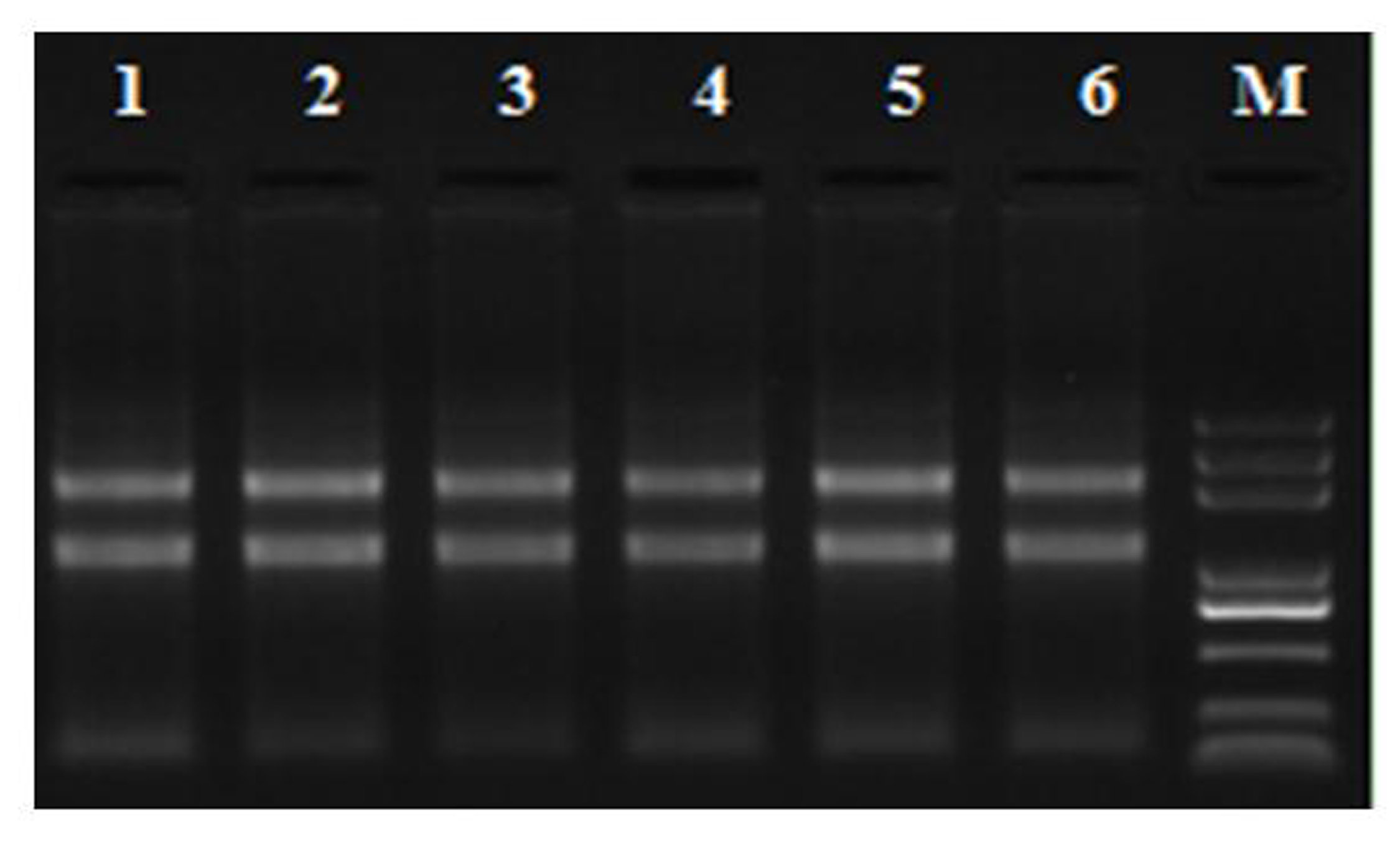

Supplement: S1 Fig — (TIF) [file pone.0191564.s001.tif]

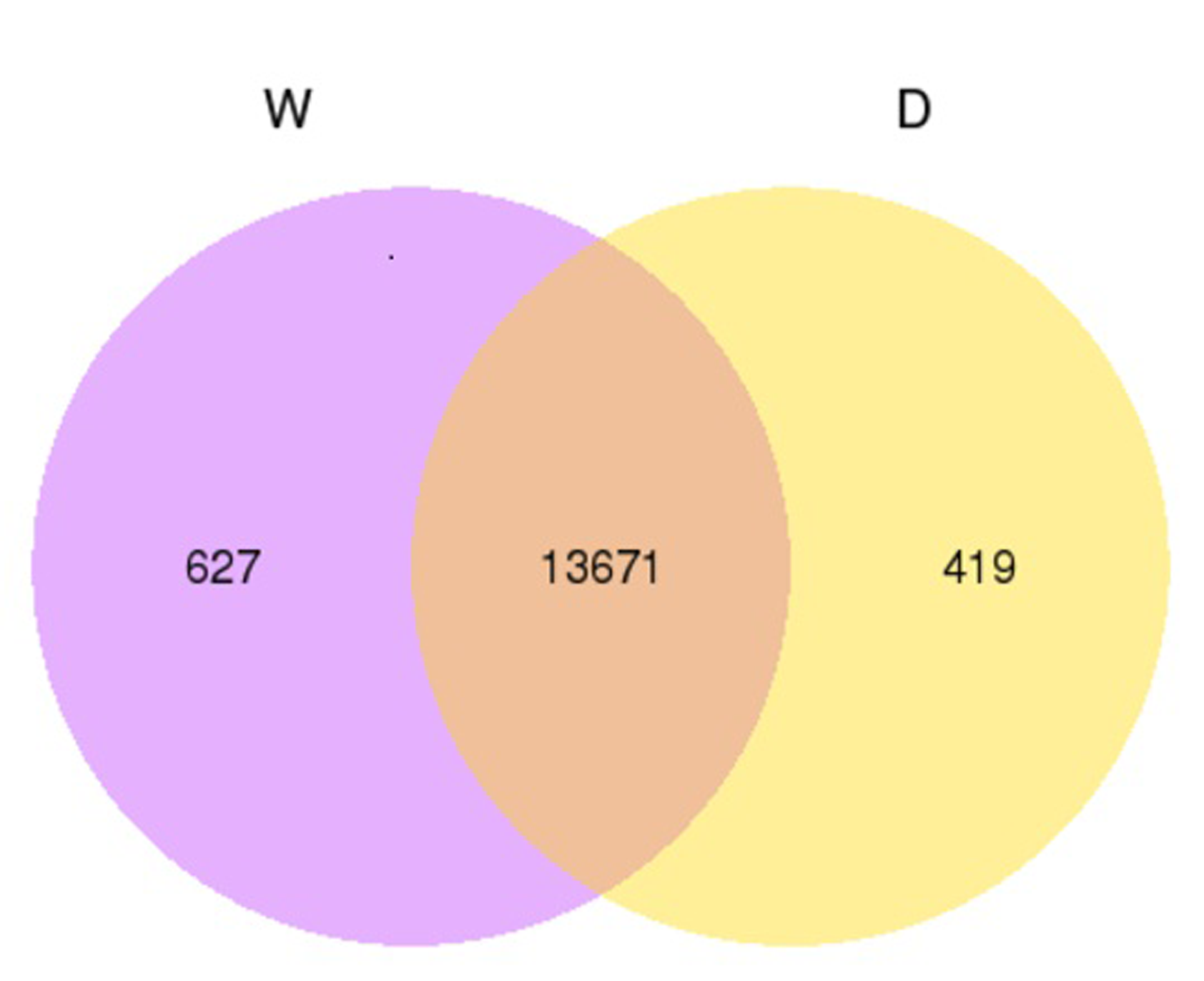

Supplement: S2 Fig — (TIF) [file pone.0191564.s002.tif]

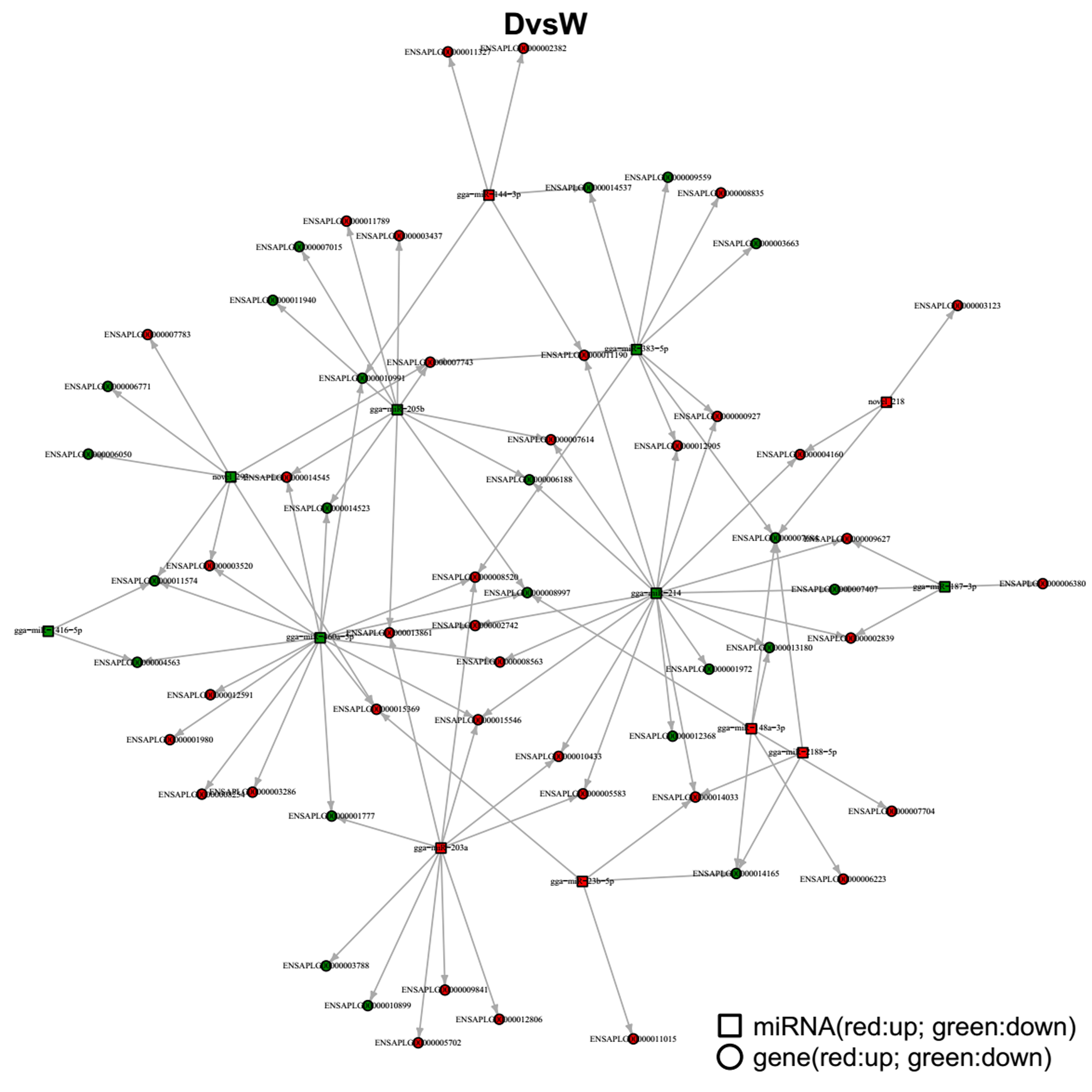

Supplement: S3 Fig — Note: Square nodes and round nodes in the figure represent miRNAs and genes, respectively. The line represents the correlation between the differentially expressed miRNA and target genes. Red and green represent up and down differential expressed miRNAs and mRNAs respectively. (TIF) [file pone.0191564.s003.tif]

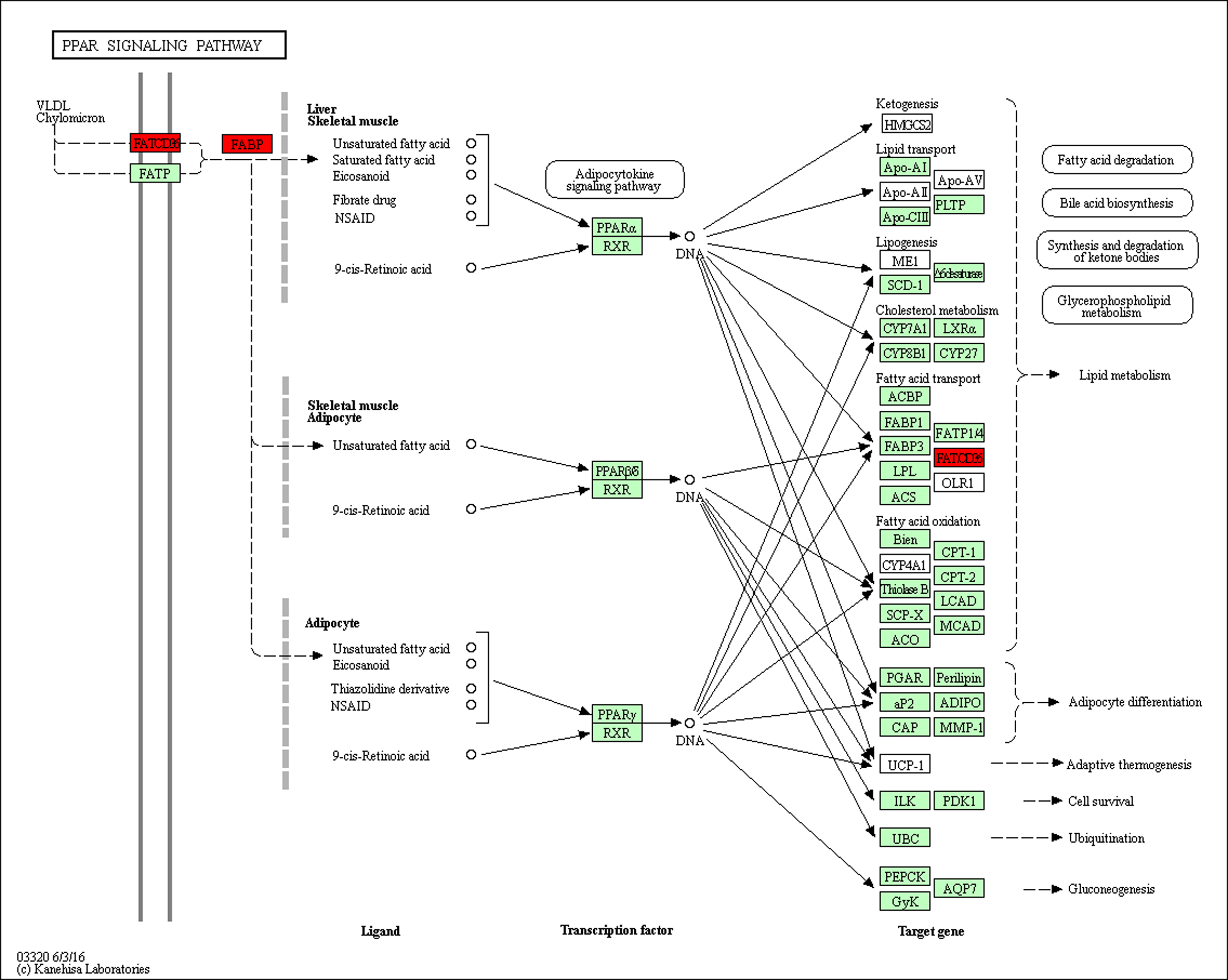

Supplement: S4 Fig — Note: red represents target genes that are enriched in the pathway. (TIF) [file pone.0191564.s004.tif]
